# Effect of Ethanol on the Textural Properties of Whey Protein and Egg White Protein Hydrogels during Water-Ethanol Solvent Exchange

**DOI:** 10.3390/molecules25194417

**Published:** 2020-09-25

**Authors:** Christian Kleemann, Joël Zink, Ilka Selmer, Irina Smirnova, Ulrich Kulozik

**Affiliations:** 1 Chair of Food and Bioprocess Engineering, Technical University of Munich, Weihenstephaner Berg 1, 85354 Freising, Germany; joel.zink@tum.de (J.Z.); ulrich.kulozik@tum.de (U.K.); 2 Laboratory of Food Process Engineering, ETH Zürich, Schmelzbergstrasse 9, 8092 Zürich, Switzerland; 3 Institute of Thermal Separation Processes, Hamburg University of Technology, Eißendorfer Straße 38, 21073 Hamburg, Germany; ilka.selmer@tuhh.de (I.S.); irina.smirnova@tuhh.de (I.S.)

**Keywords:** ethanol-protein interaction, gel stability, gel shrinkage, alcogel, aerogel

## Abstract

This study aims at investigating the effect of ethanol (EtOH) on the textural properties of whey protein and egg white protein hydrogels. The hydrogels were produced by thermally induced gel formation of aqueous protein solutions. The water contained in the gel network was subsequently exchanged by EtOH to assess structural changes upon exposure of hydrogels to ethanolic aqueous phases. The textural properties of the hydrogel and alcogel samples were analyzed by uniaxial compression tests. For both protein sources, the hardness increased exponentially when pH and EtOH concentration were increased. This increase correlated with a shrinkage of the gel samples. The gel texture was found to be elastic at low EtOH concentrations and became stiff and hard at higher EtOH concentrations. It was found that the solvent exchange influences the ion concentration within the gels and, therefore, the interactions between molecules in the gel structure. Non-covalent bonds were identified as substantially responsible for the gel structure.

## 1. Introduction

The effect of ethanol (EtOH) on proteins in solution as well as on resulting protein or polysaccharide gels has been studied by various works for several reasons. Applications, where an understanding of changes induced by EtOH on proteins is of relevance, are the disinfecting properties of EtOH, where it is highly effective against enveloped viruses [1]. The use of alginate gels as super-absorbent for EtOH containing aqueous solutions has been studied by Moe et al. [2]. In food products, protein gels may be used itself as semi-solid food containing alcohol or as a carrier system for microorganisms in fermentation processes producing EtOH, where a change of the gel structure induced by variable EtOH weight fractions may have negative effects on the fermentation process [3].

A protein hydrogel can be described as a porous matrix and networked structure completely soaked with water as a gel solvent. Hydrogels also function as precursors of aerogels, following solvent exchange by ethanol (EtOH) and supercritical CO_2_ extraction [4,5,6,7]. The dry protein network structure has an extremely high inner porosity and high specific inner surface area [4]. As a carrier system, this surface area may be impregnated with large amounts of a valuable component requiring protection against adverse conditions in the environment [8]. Before hydrogels can be converted into an aerogel, exchanging the contained water entirely by CO_2_-soluble EtOH is necessary [6]. However, the gel is postulated to undergo structural changes during the solvent exchange step of the water with a CO_2_-soluble solvent like EtOH and the scCO_2_-drying step [9], but it is unclear whether the structural changes occur on the way from the hydrogel to the alcogel or from the alcogel to the aerogel. The effect of EtOH on stabilizing binding forces in the hydro- and alco-gel respectively, is not well understood [10,11].

Heat-denatured protein gels often obtain their structural stability through covalent bonds in the form of disulfide bridges or non-covalent molecular interactions (electrostatic and hydrophobic interactions, hydrogen bonds), depending on the nature of the protein [12]. Non-covalent molecular interactions are particularly influenced by the solvent exchange. EtOH has a much lower relative permittivity than water, which influences electrostatic interactions [13]. In addition, water interacts much stronger with the nonpolar solvent than with the proteins [14]. A denaturing effect, which puts the protein molecules in the molten globule intermediate state has also been reported for organic solvents [15]. Therefore, protein molecules dissolved in aqueous solution aggregate and subsequently, precipitate, when EtOH is added to the solution, because hydrophobic interactions increase.

Reports related to the effect of EtOH on proteins in solution or also on polysaccharides already in gel state at various environmental conditions [1,2,16,17] do not allow for a prediction of the behavior of milk protein and hens’ egg white protein gels produced by thermally induced gel formation. The purpose of this study therefore was to assess the effect of EtOH on such gels after the aqueous protein solution has been gelled by heat treatment. Other studies have focused on the effect of EtOH on proteins in solution, prior to gel formation. The methods used to assess potential structural or molecular changes, like circular dichroism [1,15], cannot be applied to ready set gels, because the protein molecules are bound to the protein network structure. The hypothesis was that certain EtOH concentrations and pH levels can be expected to lead to different properties of the alcogels due to the impact of EtOH on existing and developing non-covalent bonds. It was expected that egg white protein (EWP) and whey protein isolate (WPI) yield different gel properties, because the individual proteins contained in the complex EWP and WPI systems have different properties and, therefore, should react differently when the already structured proteins in gel state, thermally produced in environments of different pH, are immersed in EtOH. The aim and the point of difference of this study was to extend the existing knowledge on the presence of EtOH prior to and during gel formation. Furthermore, the fate of ions contained within the protein networks upon solvent exchange was assessed. Knowledge about potential shifts in the ion concentration was expected to yield novel insights regarding ionic retention or washing out effects during the solvent exchange. Protein-protein interactions are known to depend on the polarity of the solvent and their ionic environment. With the results obtained, a gap in knowledge is intended to be closed since changes observed in the final aerogel were attributed to the overall process, not differentiating between changes from the protein hydro- to the alco-gel.

The approach was as follows: Heat-denatured WPI and EWP hydrogels of different pH were immersed in ethanolic aqueous solutions containing EtOH concentrations of 60–100%. Textural changes were analyzed with uniaxial compression tests to measure the sample height, the hardness and elasticity of the gels. To determine the amounts of ions dissolved from the gel matrix during solvent exchange, a method was developed to measure the cation content in the surrounding exchange solution using high-pressure liquid chromatography (HPLC).

## 2. Results

### 2.1. Shrinkage of Protein Hydrogel Spheres as A Function of Ethanol Concentration and pH during Thermal Gel Formation

The solvent exchange was conducted by immersing the gel in an aqueous EtOH solution with a concentration of 60% to 100% EtOH. The exchange solution was renewed four times after at least 4 h, i.e., when equilibrium was reached. A constant EtOH weight fraction in the surrounding solution was taken as an indicator for a completed solvent exchange. In all experiments, the final EtOH concentration was at least 99% of the target concentration.

**Figure 1:**
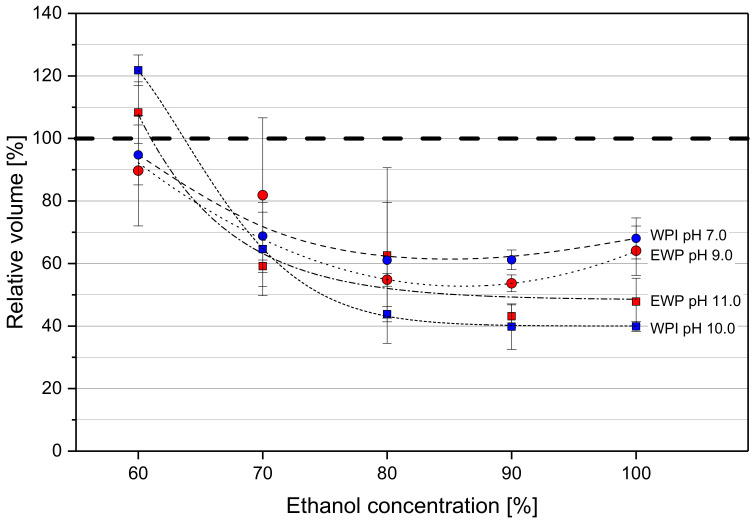
Relative volume of gel sphere samples after immersion in the respective aqueous ethanol (EtOH) solution. WPI gel samples are marked in blue, and EWP samples are red. Circles represent the native pH, squares represent alkaline conditions. Dashed lines are a guide to the eye.

The volumetric changes were determined by measuring the diameter of the spherical gel samples in order to investigate swelling or shrinkage of the gels. The volume of the alcogels after the solvent exchange was related to the volume of the respective hydrogel samples. In Figure 1, the relative sample volume is depicted as a function of EtOH weight fraction in the aqueous solution.

EtOH weight fractions around 60% were determined as being near the threshold between swelling and shrinkage of the gel spheres, independent of the type of protein and pH prior to gel formation. At 60% EtOH, gels revealed slight shrinkage at their native pH, whereas gels with an alkaline pH even gained some volume. Immersing the same gels in deionized water led to extensive swelling with a volume increase of 236% ± 41% (EWP) and 376% ± 15% (WPI) at native pH. The swelling of gels formed at alkaline pH was even more pronounced, with 873% ± 39% (EWP) and 644% ± 99% (WPI).

EtOH concentrations above 60% reduced the volume of the gel spheres. The shrinkage was more pronounced for gel spheres formed in alkaline environment. The final relative volume of gels at the protein solution’s native pH was around 65%. The most extensive shrinkage was observed for WPI gels with pH 10.0. At 100% EtOH, their size was only 40% of their initial volume. The data points indicate that extensive shrinkage happens up to an EtOH weight fraction of 80%. Higher EtOH concentrations did not lead to further shrinkage. The higher standard deviations up to 80% EtOH can be explained by the very soft gel structure, which causes more variability in measuring the diameter. The harder gel structures at higher EtOH concentrations reduce the variability. The volume changes observed were in the first place determined by the pH of the protein solution prior to gel formation. An effect of the type of protein on the volume change was not observed.

### 2.2. Hardening of Gel Spheres Induced by EtOH

**Figure 2:**
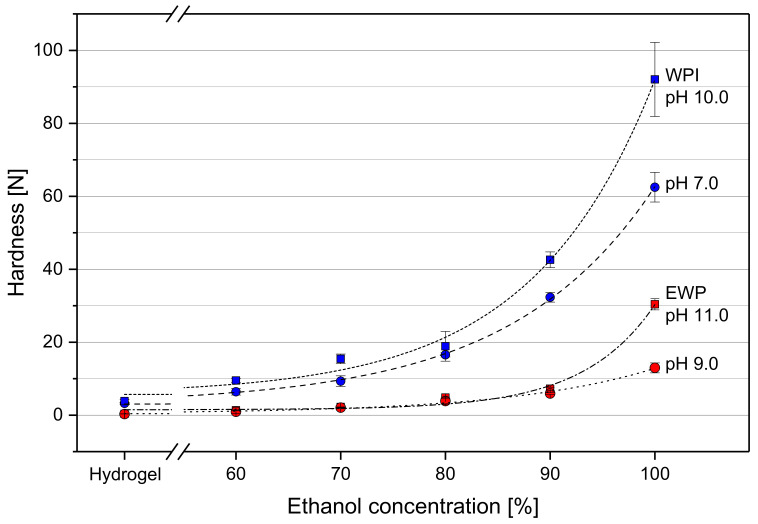
Hardness as the necessary force for compression of the gel spheres to 60% of their original height as a function of EtOH weight fraction in the gel after solvent exchange. WPI gel samples are marked in blue, EWP samples are in red. Circles represent the native pH, squares represent alkaline conditions. Dashed lines were added as guide to the eye.

The solvent exchange from water to EtOH had an evident impact on the hardness of the gel spheres. No fracture peaks were detected by the texture analyzer for all tested protein gels when the samples were compressed by 60% of their original height. This was true for all tested pH values and was independent of EtOH concentration. The necessary force for compression as a function of EtOH weight fraction in the surrounding solution at the end of the solvent exchange is depicted in Figure 2 for varying types of protein and pH values.

The untreated hydrogels were soft and could be deformed with a relatively low uniaxial compression force. The absolute forces needed to compress WPI hydrogels were 3.22 ± 0.34 N and 3.84 ± 0.44 N for pH 7.0 and pH 10.0, respectively. These values are about ten times higher in comparison to EWP gels, where 0.29 ± 0.02 N were required for pH 9.0 and 0.35 ± 0.01 N for pH 11.0.

Independently of protein type and pH, an exponential increase in compression force was detected in all cases with increasing EtOH concentration. This effect was more pronounced for gels formed in alkaline environment. After the solvent exchange with an aqueous solution with 60% EtOH (*w/w*), the gels required only slightly more force for the same deformation compared to the hydrogels. A moderate increase of the necessary compression force was needed up to 80% EtOH. At 100% EtOH, the necessary compression force was at least ten times higher compared to an EtOH concentration of 60%. WPI gels reacted stronger to the presence of EtOH than EWP gels. The necessary compression force was about 3 to 4 times higher for WPI gels.

### 2.3. Effect of EtOH on Elasticity of Gel Spheres

**Figure 3:**
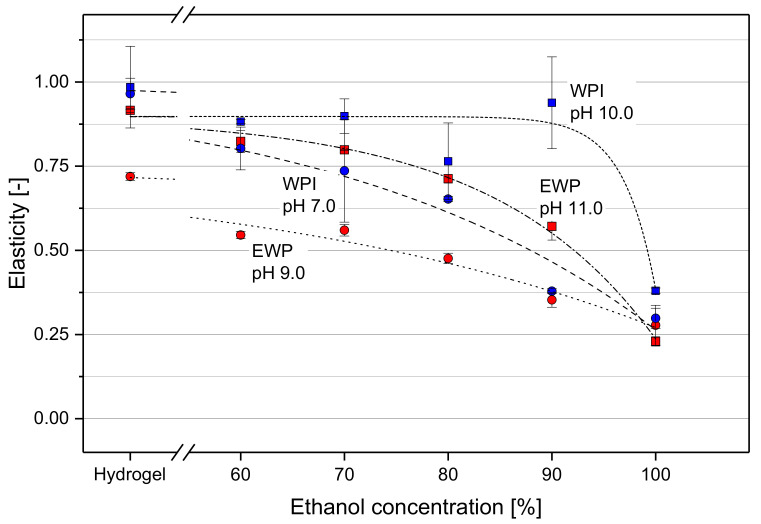
Elasticity of gel spheres as a function of EtOH weight fraction in the gel after solvent exchange. Blue symbols represent WPI gels, red symbols EWP gels. Circles are for native pH, squares for alkaline pH values. Dashed lines are a guide to the eye, only.

The elasticity of the gels as a function of EtOH weight fraction, protein type and pH at gel formation was determined by a texture profile analysis (TPA). The values of elasticity are shown in Figure 3 as the ratio of the height of the sample at the beginning of the second to the first compression cycle of the TPA. An elasticity value of 1.00 describes a sample that completely regains its original height between the two compression cycles. This would indicate that the cross-links between the molecules within the gel network are not affected during compression and are able to force the gel back to the state prior to deformation before the second compression cycle begins.

Nearly full elastic recovery was observed for WPI hydrogels at pH 7.0 and 10.0, and EWP hydrogels at pH 11.0. Their textural elasticity values were between 0.91 and 0.99, which shows that a strain of 60% was within the range of elastic deformation for those gels. Only the pH 9.0 EWP hydrogel showed a substantially lower elasticity, with only 0.72. The measured elasticity was lower for all tested types of gel when the solvent exchange to EtOH was conducted and deformation became plastic. This was especially the case for the pH 9.0 EWP samples. With increasing EtOH concentration, the elasticity dropped down to about 0.25. This was also the case for the pH 11.0 EWP gel and the pH 7.0 WPI gel, but the drop to lower elasticity values occurred at higher EtOH concentrations. The high elastic property was best maintained for WPI gels with pH 10.0. An EtOH concentration above 90% was necessary to decrease the elasticity of those gels. At 100% EtOH, a sudden decrease of elasticity was observed with a final elasticity of 0.37, which is in the range of the other three tested gels.

### 2.4. Interaction of Ions Contained in the Protein Gel with the Exchange Solution

The ion concentration within a protein gel influences the interactions between the proteins and has an effect on the gel hardness and elasticity. It was analyzed whether ions dissolve in the exchange solution or remain in the protein gel, which is an indirect measure of the ion concentration within the gel. The amounts of ions in the exchange solutions relative to the ones in the protein gel prior to the solvent exchange are listed in Table 1. The intention was to understand how the solvent exchange influences the ion concentration within the gel and to identify differences between WPI and EWP gels. Both gels were analyzed at their native pH of 7.0 (WPI) and 9.0 (EWP).

All exchange solutions were ion-free at the beginning of the solvent exchange. Therefore, all ions detected in the exchange solution after the solvent exchange diffused from the gel into the surrounding liquid. When the gel spheres were immersed in deionized water, over 80% of the ions initially added with the gel spheres were found in the exchange solution. The ions interacted stronger with deionized water as a solvent than with the proteins in the gel. A stronger interaction was observed between WPI gel proteins and Ca^2+^ ions. Only a little above 30% of the Ca^2+^ ions diffused from the gel into the exchange solution. With an increasing EtOH-concentration, the concentration of cations found in the solvent after the exchange decreased. In the case of WPI gels, nearly all ions remained within the gel when the solvent was 100% EtOH. The interactions between the ions and the proteins are stronger than with the solvent. The same trend was observed for EWP gels. Only the results of Ca^2+^ ions (Table 1, last column) do not strictly follow this trend. The generally very low concentration of Ca^2+^ ions in EWP (less than 1% of Na^+^-concentration) brought along an uncertainty in the analytical values. However, the amounts of ions solubilized from the EWP gels in the ethanolic solvent was higher compared to WPI gels. The results indicate that the ionic environment within EWP gels is more strongly influenced by a solvent exchange compared to WPI gels, because more ions were detected in the exchange solution.

## 3. Discussion

Shrinkage on one hand and harder gels on the other hand upon solvent exchange were observed for all tested gel samples, starting with an EtOH weight fraction of 60%. Extensive swelling was observed in deionized water. Therefore, it can be assumed that around an EtOH concentration of 60%, the attracting forces, namely hydrophobic interactions and hydrogen bonds, start to compensate the repulsive forces, mainly electrostatic repulsion, between molecules in the protein gel structure. Those non-covalent interactions are predominantly responsible for the hardness of heat-induced protein gels [12]. This effect of EtOH on protein gels from whey or egg white, which were gelled prior to contact with EtOH, has not been investigated so far. A comparable distinct threshold between swelling and shrinkage has been reported for polysaccharide gels from gelatin, Na-alginate and κ-carrageenan in the literature [2,3,17]. Yoshizawa et al. [1] reported a precipitation of bovine serum albumin molecules in solution at or above an EtOH weight fraction of 60%. An EtOH fraction of 50% (*v/v*) can also be used to induce gelation of whey protein solutions without heat treatment [18]. An increasing hardness of WPI gels for increased EtOH weight fractions was also measured by Zirbel and Kinsella [19]. In their study, the researchers added EtOH to WPI solutions before heat-induced gelation. In comparison to the results presented here, the intermolecular interactions were influenced by EtOH prior to forming covalent and non-covalent bonds. Uversky et al. [15] reported that EtOH induces a similar molten globule state as thermal heat treatment, which leads to exposed hydrophobic regions of the protein, usually buried within the globular structure. Hydrophobic interactions then lead to aggregation. The structural changes on secondary protein structure or the electrostatic interactions, which have been observed for whey and egg white proteins in ethanolic solution [15,20,21,22], seem to apply in a similar way to the heat set whey and egg white gels in this study.

EtOH with its low relative permittivity leads to stronger electrostatic interactions between the proteins, which have a negative net charge, due to a pH above the isoelectric point (pI) during gel formation, and the ions within the solvent [23]. The pI of proteins contained in EWP ranges between 4.1 and 10.7, whereas the proteins accounting for over 75% of the total dry mass have a pI of 4.1 to 6.1. [24]. The pI of proteins contained in WPI systems ranges between 4.2 and 8.8, and α-lactalbumin and β-lactoglobulin, which account for over 70% of the total dry mass, have a pI of 4.2 to 5.1 [25]. Due to the reduced relative permittivity, the counter-ions to the protein charges lose their mobility and accumulate at charged amino acid side chains, and dipole–dipole interactions occur, as proposed by Khokhlov et al. [26], while repulsive electrostatic forces are diminished. This leads to a denser packing of the molecules and the formation of new and stronger hydrophobic interactions and hydrogen bonds, resulting in shrinkage and hardening of the gel structure. The results in Table 1 showed that without EtOH, most cations diffused from the gel matrix into the surrounding water. This reduced the ion concentration within the gel. This diminished the shielding effect on repulsive forces between equally charged protein molecules. The increasing repulsive forces in the gel network contribute to the extensive swelling behavior of protein gels in aqueous solutions in addition to the mechanisms described by Betz et al. [27] and Gunasekaran et al. [28]. With increasing EtOH concentration, the concentration of cations found in the exchange solution was reduced. The results indicate that the ions interact more strongly with the proteins at higher EtOH concentrations than with the solvent. A major contribution to this effect was also seen in the very low solubility of ions in EtOH-containing solvents [29]. The ions remain within the gel and, in addition, their effect on protein–protein interactions is enhanced by the low relative permittivity of EtOH. It is known that a high ion concentration supports the aggregation of proteins [30,31]. The fate of ions within the gel structure during solvent exchange with EtOH, therefore, supports the shrinkage and hardening of the protein gels.

A special role can be attributed to Ca^2+^ in WPI gels, where the ions interact very strongly with the proteins, thus, substantially less Ca^2+^ was found in the surrounding water. This can be explained by the divalent nature of Ca^2+^. This enables this cation to form strong Ca-bridges between the negatively charged amino acid residues [32].

More cations have been found in the exchange solution of EWP gels (see Table 1). This would generally lead to a reduction of the shielding effect and the repulsive electrostatic forces of the negatively charged molecules have a larger effect, which, in turn, leads to swelling and softening of the gels. However, shrinkage and hardening were still observed for EWP gels (Figure 1 and Figure 2). The denaturing effect of EtOH and its lower relative permittivity, which strengthens the interactions between molecules and remaining ions, seem to outweigh the reduced ion concentration within the gel.

Shrinkage and hardening of the gel spheres were more pronounced when the hydrogels were formed in strong alkaline environment (Figure 1 and Figure 2). This was attributed to the more ordered filamentous gel structure in strong alkaline conditions. Due to the high negative net charge of protein molecules in an environment far more alkaline than the pI, an aggregation of the protein molecules takes place in long chain-like structures. The number of covalent disulfide bonds increases due to a higher reactivity of thiol groups at alkaline pH. The number of non-covalent bonds, like hydrophobic interactions or hydrogen bonds, decreases due to the strong repulsive electrostatic interactions. The final gel has a structure composed of stranded aggregates [33]. A random aggregation of the molecules is reduced, as it occurs with a pH closer to the pI during gel formation due to the more neutral net charge. This aggregation takes place when the non-covalent bonds are enhanced by the addition of EtOH. In addition, the particles of a particulate gel, generated at a gel forming pH closer to the pI [34], prevent a packing as dense as in the alkaline gel structure. The particles function as spacers in the gel network structure and larger cavities remain in the structure. This yields less shrinkage of the gels and a softer gel structure at a high EtOH concentration for gels with a lower pH. It is known that EtOH may raise the pI of proteins, because it slightly raises the pK_a_ values of amino acid side chains [35,36]. The pH of the gels, however, was in an alkaline region with an explicit gap to the pI. Due to the complex protein systems of WPI and EWP, containing a large number of proteins, a discussion of single pK_a_ values is not conducive. The results indicate that the pH during gel formation, which influences the net charge of the proteins and, therefore, a different gel structure, has a clear effect on the changes in gels induced by EtOH. This can be of high relevance in applications where protein gels are used as a carrier system for active microorganisms in continuous fermentation processes of EtOH. The dense structure of gels formed in very alkaline conditions would diminish the diffusion of substrate and product. To tailor gel properties to certain requirements, an adequate EtOH concentration and pH could be chosen based on the results presented here.

Furthermore, it was observed that the increase of hardness, determined for an increasing EtOH concentration, was not linear but rose exponentially (Figure 2). This was not expected from the volumetric change data shown in Figure 1. There was no considerable change of volume observed at EtOH weight fractions above 80%. This would have suggested that there would also be no more change in hardness at higher EtOH concentrations. In contrast, the hardness at 100% EtOH was three- to six-fold higher than at 80%. This is a clear indication that the gain in hardness is not a direct result of the loss of volume, attended by a higher density of the gel structure. It is rather a result of enhanced non-covalent cross-linking and, thus, of a restructuring of the protein network.

A characteristic of gels is that they enclose a large amount of solvent. This permits a high structural flexibility of the molecules despite being less mobile in the protein network. With the results presented, it can therefore be stated that the effects of EtOH on gel hardness observed by Zirbel and Kinsella [19], who added EtOH prior to heat set gelation, or Dufour et al. [18] and Yao et al. [20], who induced protein gelation by the addition of EtOH, also apply to hydrogels after heat set gelation. The high elasticity of hydrogels (Figure 3) gives an impression of this structural flexibility. The hydrogels regained their original height after deformation, which is expressed in an elasticity value close to 1.0. This applies to gels formed in a strong alkaline environment in particular. This is attributed to an easy displacement of the filamentous strands against each other, which, in addition, are negatively charged. The repulsive forces between these equally charged molecules allow deformation without irreversible destruction of the gel network. Furthermore, the alkaline pH promotes thiol-disulfide exchange reactions [37], which contribute to the gel structure and stability. Those covalent bonds do not break up during deformation. Therefore, an important role was attributed to those covalent bonds in helping the gel body to regain its original size after compression of hydrogels. The lowest elasticity values were measured for EWP hydrogels at pH 9.0. This was attributed to the more particulate structure of these gels, which becomes evident by their opaque appearance, as discussed above. The dominating intermolecular forces are of hydrophobic character, hydrogen bonds and dipole-dipole and electrostatic interactions. The less alkaline environment led to a lower reactivity of thiol groups and less covalent bonds developed. The non-covalent cross-links in the more particulate network structure broke during deformation, and this destruction is not reversible.

With an increasing EtOH concentration, the deformation of all tested gels becomes more permanent. The non-covalent bonds stabilizing the gel become stronger, due to the influence of EtOH on protein-protein interactions. They rearrange between displaced protein strands after application of mechanical stress on the hydrogel. The newly created non-covalent cross-links hold the gel in the deformed position, which results in low elasticity values.

The WPI pH 10.0 gels only lost their elasticity at very high EtOH concentrations. The strong electrostatic repulsion due to the high negative net charge in alkaline environment prevented extensive formation of new non-covalent molecular cross-links up to 90% EtOH. Even at this high EtOH concentration, the protein strands evidently permitted a displacement parallel to each other during compression without irreversible destruction of the gel network. The network components were forced back to their original position after the deformation cycle. Only at an EtOH concentration of 100% the interactions did become strong enough to eliminate the elastic properties almost completely. This sudden loss of elasticity coincided with an exponential increase of hardness at high EtOH weight fractions (see Figure 2).

The results presented here show that a transition from a very soft to a very hard gel occurs during a solvent exchange from a hydrogel to an alcogel. This change from a soft and elastic gel structure to a hard and robust structure was also observed in the process of creating protein aerogels [4,6], but it was unclear whether the hardening effect occurs already during solvent exchange or afterwards during scCO_2_ drying. An effect of the pH during gel formation was also observed, where the hydrogels became softer with increasing pH. The opposite was true for the corresponding aerogels, which become much harder with increasing pH [38]. Table 2 shows the stiffness (calculated as required force per distance of deformation) of the hydrogels and alcogels compared to published values for aerogels.

The values reveal that for EWP gels, the stiffness increased by a factor of up to 10^2^ from the hydrogel to the alcogel state. The stiffness was doubled at most from the alcogel to the aerogel state. This shows that a substantial part of the structural changes already occurred during the solvent exchange. A similar effect was observed with WPI. The relative effect of scCO_2_ drying is higher for WPI gels than for EWP gels, but a major effect of the solvent exchange is visible. It is undoubted that the scCO_2_ drying induces further changes because the gel changes from the typical gel character containing a liquid into a liquid-free solid material. On the insights gained in this study, we can state that the scCO_2_ process seems to be of minor effect to the shrinkage and hardening of the protein gels, but rather enforces changes, which are to a major extent induced by the solvent exchange. This can be derived from the fact that the aerogel properties resemble most of the properties already observed for the alcogel. Most changes can therefore be attributed to the solvent exchange step. This is a major difference to silica aerogels, where the gel formation is typically performed directly in methanol or ethanol and all shrinkage can be attributed to the supercritical drying step [39]. The newly formed and strengthened interactions in the gel structures due to the solvent exchange of water with EtOH are, therefore, most important for the properties of supercritically dried protein aerogels.

## 4. Materials and Methods

### 4.1. Materials

Pasteurized egg white was provided by Ovobest Eiprodukte GmbH & Co. KG (Neuenkirchen-Vörden, Germany). Whey protein isolate powder with a protein content of 94% was obtained from Davisco Foods International Inc. (Eden Prairie, MN, USA). Commercial sunflower oil was sourced from a local store (Bröckelmann + CO, Hamm, Germany). EtOH (99.95% purity) was obtained from VWR Chemicals (Leuven, Belgium). Hydrochloric acid and sodium hydroxide came from Merck KGaA (Darmstadt, Germany). Perchloric acid 60% to dissolve ions as well as sulfuric acid 0.4 mM and oxalic acid 2.3 mM as eluent for HPLC were purchased from Merck KGaA (Darmstadt, Germany). Sodium IC standard solution and potassium AAS standard solution came from Carl Roth GmbH (Karlsruhe, Germany) and calcium chloride dihydrate from Sigma-Aldrich (Saint Louis, MO, USA). All chemical agents were of analytical grade. Buffers and solutions were prepared with deionized water (Milli Q Integral 3, Merck KGaA, Darmstadt, Germany).

### 4.2. Methods

#### 4.2.1. Preparation of Spherical Protein Hydrogel Samples

**Figure 4:**
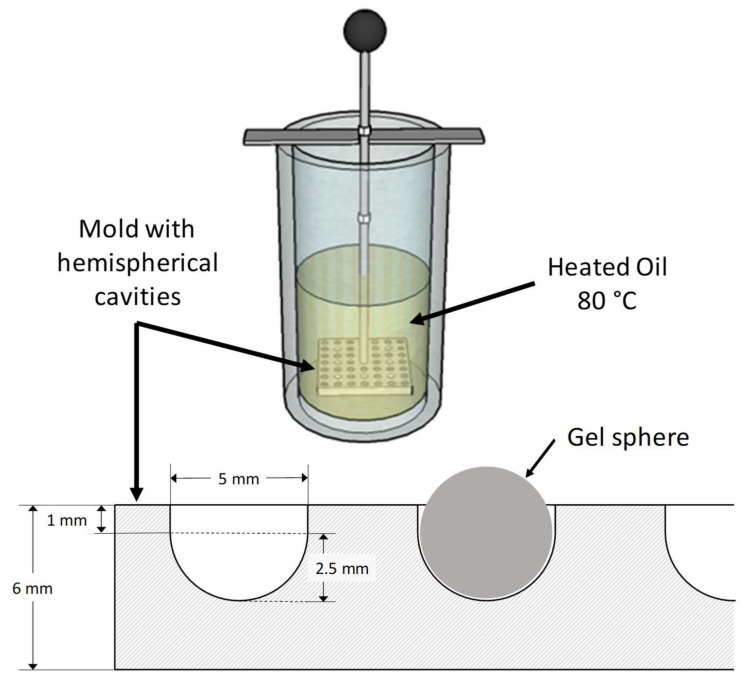
Oil bath and sectional drawing of mold for sample preparation of spherical protein gels with a diameter of 5 mm.

Protein hydrogel spheres with a diameter of 5 mm were produced using a method developed by the authors in the course of this research project. The volume of a 5 mm sphere was pipetted into a hemispherical teflon mold and immersed in heated sunflower oil for thermal gelation, as depicted in Figure 4. Supported by the mold, the interfacial tension between oil and aqueous protein solution forces the sample into a spherical shape. The method allows the production of macroscopic gel samples, under conditions as close as possible to particles produced by the emulsion gelation method. The WPI solution had a protein concentration of 20%, which is sufficient to form inherently stable hydrogels [40]. EWP hydrogels were formed with the native protein content of pasteurized egg white, determined to 10.1%. The pH of the respective protein solutions was adjusted to the specified value. The pH values chosen were the native pH of the respective protein solution (WPI: pH 7.0, EWP: pH 9.0) and the highest possible pH prior to a fast alkaline gel formation (WPI: pH 10.0, EWP: pH 11.0) The pH levels in this study always refer to the respective aqueous protein solution prior to gel formation. Hydrogel samples were stored at 4 °C in sunflower oil to prevent dehydration until further use or characterization.

#### 4.2.2. Solvent Exchange of Water by EtOH

The sphere was chosen as a sample geometry since it is the only geometry that undergoes isotropic expansion or shrinkage during swelling or de-swelling [41]. The solvent exchange with EtOH was started by removing the samples from the oil and blotting them with a lint-free paper towel. Single gel samples were transferred into 2 mL vials. The vials were then filled with 2 mL of the respective aqueous EtOH solution containing 60% to 100% EtOH (*w/w*). The vials were kept under constant agitation in order to minimize concentration gradients. For a complete solvent exchange, the samples were kept in the exchange solution for at least 4 h before the solution was exchanged, which was repeated in triplicate, according to Kleemann et al. [38]. The final EtOH content of the samples was determined indirectly with a density meter DM4100 (Anton Paar GmbH, Graz, Austria) by measuring the EtOH concentration of the solution after the respective exchange step. Samples were stored at 4 °C in the final EtOH solution until further characterization.

#### 4.2.3. Physical Characterization of Spherical Samples

Prior to structural characterization, the samples were tempered at 20 °C for 1 h. The samples were removed from the respective storage fluid, blotted with a lint-free paper towel and analyzed immediately to avoid drying up. To determine the textural properties of the gel spheres, a texture analyzer TA.XT.plus (Stable Micro Systems, Vienna Court, UK) with an acrylic cylinder probe (diameter 13 mm) was used. Pre-test speed, test speed and post-test speed of the probe were all set to 0.1 mm/s. The height of the probe at the first sign of a counteracting force was used to determine the diameter of the samples. A texture profile analysis (TPA) measurement according to Bourne [42] was conducted to measure the hardness of the samples. The hardness was defined as the force needed to deform the spherical samples by 60% of their original height. The strain of 60% was chosen because some alcogels revealed fracture peaks if a strain of 70% was exceeded. The elasticity as a measure of sample recovery after deformation was expressed as the ratio of the height of the sample at the second compression cycle to the height at the first compression cycle with 60% strain and 5 s recovery time between the cycles.

#### 4.2.4. Measurement of Dissolved Ions from Protein Gel Structure during Solvent Exchange

Gel spheres were produced according to Section 4.2.1. The WPI solution had a pH of 7, and the EWP solution had a pH of 9 prior to gel formation. Directly after sample production, 10 gel spheres were placed into 10 mL vials with 4 mL of exchange solution. The exchange solutions were: deionized water, 60% aqueous EtOH solution or 99.95% EtOH solution. The solvent exchange was then conducted during 24 h at 4 °C under constant agitation to minimize concentration gradients and microbial growth. This exchange time was chosen since it allowed the detection of the ions in each sample. Shorter exchange times and a repetitive renewal of the exchange solution, as conducted for the complete solvent exchange (Section 4.2.2), would have reduced the content of ions below the detection limit. After the exchange time, the gel samples were removed from the exchange solution, and weight and density (DM 4100, Anton Paar GmbH, Graz, Austria) of the solution were determined. Due to incompatibility of EtOH and the HPLC analysis of cations, the entire exchange solution was evaporated in a vacuum dryer (VO Cool, Memmert GmbH & Co.KG, Schwabach, Germany) overnight at 80 mbar and 50 °C. The remaining ions were then re-dissolved by the addition of 2 mL deionized water and 100 µL of 60% perchloric acid, which also dissolved and denatured residual proteins in the vials, which were inevitably found as traces of the gel spheres after the solvent exchange. After a dissolution time of 10 min, another 6 mL of deionized water was added and the samples were centrifuged with a 10 kDa centrifugal filter (VWR Centrifugalfilter 10 kDa, 500 µL, VWR International, Radnor, PA, USA) at a relative centrifugal force of 14,000× *g* during 10 min. The filtrate was diluted at a ratio of 1:1 with deionized water to perform HPLC-analysis. A PRP-X800 column for isocratic separation of mono- and di-valent cations was used to determine the ion concentration of Na^+^, K^+^ and Ca^2+^. Potassium was below the detection limit for WPI samples. The concentration determined with HPLC analysis allowed to calculate the absolute weight of ions in the vials after evaporation. Using the absolute weight of ions, the concentration of ions in the solution after the solvent exchange was calculated, using the volume of the exchange solution, determined by its mass and density.

#### 4.2.5. Determination of Extracted Ions from Protein Gel during Solvent Exchange

The ions in the precursor protein solutions, used for the hydrogel preparation, were determined by applying the same method as described in Section 4.2.4. In brief, 200 mg of the protein solutions were placed in a vacuum dryer to evaporate the water. By the addition of deionized water and perchloric acid, the ions were dissolved, and the proteins precipitated prior to centrifugation with centrifugal filters. The concentration of ions in the filtrate was determined by HPLC analysis. Using this concentration, the absolute weight of residual ions in the vials after evaporation and, hence, the concentration in the precursor protein solution was calculated. This allowed to calculate the mass of ions contained in the gel samples prior to the solvent exchange (Section 4.2.4). The percentage of extracted ions from the gel samples was calculated as the ratio of the mass of ions contained in the exchange solution and the mass of ions in the gel samples before the solvent exchange.

(1) $$Extracted\;ions\;\left[\%\right]=\frac{m_{ions\;in\;exchange\;solution}}{m_{ions\;in\;gel\;before\;solvent\;exchange}}\cdot 100$$

#### 4.2.6. Statistical Evaluation and Data Presentation

All experiments were executed at least in triplicate. The standard for texture analyzer data points in this study was the measurement of five individual samples. Error bars in the graphs represent the standard deviation. Absolute values are given as the mean value of repetitions ± standard deviation.

## 5. Conclusions

With this study, the knowledge about the effect of EtOH on proteins has been extended from proteins in solution to proteins immobilized in heat-denatured gels based on different proteins and produced at different pH conditions. In particular, we can conclude based on the results presented here, on hydrogels exposed to solvent exchange and in previous works on aerogel structures, that most of the hardening and shrinkage occurs early in the process during solvent exchange and hardly during scCO_2_ drying of alcogels.

## Figures and Tables

**Table 1:** Ions contained in exchange solution after 24 h of solvent exchange.

Exchange Solvent	WPI pH 7.0 Na^+^ (%)	WPI pH 7.0 Ca^2+^ (%)	EWP pH 9.0 Na^+^ (%)	EWP pH 9.0 K^+^ (%)	EWP pH 9.0 Ca^2+^ (%)
H_2_O	81.9 ± 4.6	32.5 ± 2.1	98.3 ± 2.8	83.5 ± 2.0	91.0 ± 21.7
EtOH 60%	35.1 ± 2.1	9.6 ± 1.0	88.3 ± 1.1	77.0 ± 1.0	14.2 ± 0.2
EtOH 100%	4.4 ± 1.1	1.2 ± 0.8	65.0 ± 1.0	57.3 ± 1.3	31.4 ± 2.8

**Table 2:** Stiffness of gels in hydrogel, alcogel and aerogel state, calculated as required force per distance of deformation.

	EWP		WPI	
Gel State	pH 9.0 (N/mm)	pH 11.0 (N/mm)	pH 7.0 (N/mm)	pH 10.0 (N/mm)
Hydrogel	0.12 ± 0.01	0.15 ± 0.00	1.11 ± 0.12	1.33 ± 0.13
Alcogel	5.6 ± 0.8	15.0 ± 0.9	25.2 ± 2.3	41.0 ± 5.7
Aerogel	12.5 ± 2.9 ^1^	21.6 ± 1.6 ^1^	148.9 ± 16.9 ^2^	180.5 ± 13.5 ^2^

^1^ Selmer et al. [6], ^2^ Kleemann et al. [38].

## References

[1] Yoshizawa S., Arakawa T., Shiraki K. (2014). Dependence of ethanol effects on protein charges. Int. J. Biol. Macromol..

[2] Moe S.T., Skjaak-Braek G., Elgsaeter A., Smidsroed O. (1993). Swelling of covalently crosslinked alginate gels: Influence of ionic solutes and nonpolar solvents. Macromolecules.

[3] Sason G., Nussinovitch A. (2018). Characterization of κ-carrageenan gels immersed in ethanol solutions. Food Hydrocoll..

[4] Betz M., García-González C.A., Subrahmanyam R., Smirnova I., Kulozik U. (2012). Preparation of novel whey protein-based aerogels as drug carriers for life science applications. J. Supercrit. Fluids.

[5] García-González C.A., Alnaief M., Smirnova I. (2011). Polysaccharide-based aerogels—Promising biodegradable carriers for drug delivery systems. Carbohydr. Polym..

[6] Selmer I., Kleemann C., Kulozik U., Heinrich S., Smirnova I. (2015). Development of egg white protein aerogels as new matrix material for microencapsulation in food. J. Supercrit. Fluids.

[7] Stergar J., Maver U. (2016). Review of aerogel-based materials in biomedical applications. J. Sol.-Gel Sci. Technol..

[8] Selmer I., Karnetzke J., Kleemann C., Lehtonen M., Mikkonen K.S., Kulozik U., Smirnova I. (2019). Encapsulation of fish oil in protein aerogel micro-particles. J. Food Eng..

[9] Takeshita S., Sadeghpour A., Malfait W.J., Konishi A., Otake K., Yoda S. (2019). Formation of Nanofibrous Structure in Biopolymer Aerogel during Supercritical CO2 Processing: The Case of Chitosan Aerogel. Biomacromolecules.

[10] Subrahmanyam R., Gurikov P., Dieringer P., Sun M., Smirnova I. (2015). On the Road to Biopolymer Aerogels—Dealing with the Solvent. Gels.

[11] Gurikov P., Subrahmanyam R., Griffin J.S., Steiner S.A., Smirnova I. (2019). 110th Anniversary: Solvent Exchange in the Processing of Biopolymer Aerogels: Current Status and Open Questions. Ind. Eng. Chem. Res..

[12] Havea P., Watkinson P., Kuhn-Sherlock B. (2009). Heat-induced whey protein gels: Protein-protein interactions and functional properties. J. Agric. Food Chem..

[13] Mohsen-Nia M., Amiri H. (2013). Measurement and modelling of static dielectric constants of aqueous solutions of methanol, ethanol and acetic acid at T = 293.15 K and 91.3 kPa. J. Chem. Thermodyn..

[14] Løkra S., Helland M.H., Claussen I.C., Strætkvern K.O., Egelandsdal B. (2008). Chemical characterization and functional properties of a potato protein concentrate prepared by large-scale expanded bed adsorption chromatography. Lwt-Food Sci. Technol..

[15] Uversky V.N., Narizhneva N.V., Kirschstein S.O., Winter S., Löber G. (1997). Conformational transitions provoked by organic solvents in β-lactoglobulin: Can a molten globule like intermediate be induced by the decrease in dielectric constant?. Fold. Des..

[16] Cassanelli M., Norton I., Mills T. (2017). Effect of alcohols on gellan gum gel structure: Bridging the molecular level and the three-dimensional network. Food Struct..

[17] Boral S., Gupta A.N., Bohidar H.B. (2006). Swelling and de-swelling kinetics of gelatin hydrogels in ethanol-water marginal solvent. Int. J. Biol. Macromol..

[18] Dufour E., Robert P., Renard D., Llamas G. (1998). Investigation of β-Lactoglobulin Gelation in Water/Ethanol Solutions. Int. Dairy J..

[19] Zirbel F., Kinsella J.E. (1988). Effects of thiol reagents and ethanol on strength of whey protein gels. Food Hydrocoll..

[20] Yao L., Jiang A., Chen L. (2020). Characterization of ethanol-induced egg white gel and transportation of active nutraceuticals. Lwt-Food Sci. Technol..

[21] Hirota-Nakaoka N., Goto Y. (1999). Alcohol-induced denaturation of β-lactoglobulin: A close correlation to the alcohol-induced α-helix formation of melittin. Bioorganic Med. Chem..

[22] Yoshikawa H., Hirano A., Arakawa T., Shiraki K. (2012). Effects of alcohol on the solubility and structure of native and disulfide-modified bovine serum albumin. Int. J. Biol. Macromol..

[23] Dufour E., Haertl’ T. (1990). Alcohol-induced changes of β-lactoglobulin-retinol-binding stoichiometry. Protein Eng. Des. Sel..

[24] Mine Y. (1995). Recent advances in the understanding of egg white protein functionality. Trends Food Sci. Technol..

[25] Farrell H.M., Jimenez-Flores R., Bleck G.T., Brown E.M., Butler J.E., Creamer L.K., Hicks C.L., Hollar C.M., Ng-Kwai-Hang K.F., Swaisgood H.E. (2004). Nomenclature of the Proteins of Cows’ Milk—Sixth Revision. J. Dairy Sci..

[26] Khokhlov A.R., Philippova O.E., Sitnikova N.L., Starodubtsev S.G. (1995). Supramolecular structures in polyelectrolyte gels. Faraday Disc..

[27] Betz M., Hörmansperger J., Fuchs T., Kulozik U. (2012). Swelling behaviour, charge and mesh size of thermal protein hydrogels as influenced by pH during gelation. Soft Matter.

[28] Gunasekaran S., Ko S., Xiao L. (2007). Use of whey proteins for encapsulation and controlled delivery applications. J. Food Eng..

[29] Pinho S.P., Macedo E.A. (2005). Solubility of NaCl, NaBr, and KCl in Water, Methanol, Ethanol, and Their Mixed Solvents. J. Chem. Eng. Data.

[30] Koseki T., Kitabatake N., Doi E. (1989). Irreversible thermal denaturation and formation of linear aggregates of ovalbumin. Food Hydrocoll..

[31] Shimada K., Cheftel J.C. (1988). Texture characteristics, protein solubility, and sulfhydryl group/disulfide bond contents of heat-induced gels of whey protein isolate. J. Agric. Food Chem..

[32] Petit J., Herbig A.-L., Moreau A., Delaplace G. (2011). Influence of calcium on β-lactoglobulin denaturation kinetics: Implications in unfolding and aggregation mechanisms. J. Dairy Sci..

[33] Doi E., Kitabatake N. (1989). Structure of glycinin and ovalbumin gels. Food Hydrocoll..

[34] Handa A., Takahashi K., Kuroda N., Froning G.W. (1998). Heat-induced Egg White Gels as Affected by pH. J. Food Sci..

[35] Jukes T.H., Schmidt C.L.A. (1934). The apparent dissociation constants of certain amino acids and related substances in water-ethanol mixtures. J. Biol. Chem..

[36] Gelsema W.J., De Ligny C.L., Van der Veen N.G. (1979). Isoelectric points of proteins, determined by isoelectric focusing in the presence of urea and ethanol. J. Chromatogr. A.

[37] Van der Plancken I., Van Loey A., Hendrickx M.E.G. (2005). Changes in sulfhydryl content of egg white proteins due to heat and pressure treatment. J. Agric. Food Chem..

[38] Kleemann C., Selmer I., Smirnova I., Kulozik U. (2018). Tailor made protein based aerogel particles from egg white protein, whey protein isolate and sodium caseinate: Influence of the preceding hydrogel characteristics. Food Hydrocoll..

[39] Maleki H., Durães L., Portugal A. (2014). An overview on silica aerogels synthesis and different mechanical reinforcing strategies. J. Non-Cryst. Solids.

[40] Betz M., Kulozik U. (2011). Whey protein gels for the entrapment of bioactive anthocyanins from bilberry extract. Int. Dairy J..

[41] Kurnia J.C., Birgersson E., Mujumdar A.S. (2012). Finite deformation of fast-response thermo-sensitive hydrogels—A computational study. Polymer.

[42] Bourne M.C. (2002). Food Texture and Viscosity. Concept and Measurement.

